# Gait Pattern, Impact to the Skeleton and Postural Balance in Overweight and Obese Children: A Review

**DOI:** 10.3390/sports6030075

**Published:** 2018-07-31

**Authors:** Nili Steinberg, Dan Nemet, Michal Pantanowitz, Alon Eliakim

**Affiliations:** 1 Wingate College of Physical Education and Sports Sciences, Wingate Institute, Netanya 4290200, Israel; michalmirochnik@gmail.com; 2 Child Health and Sport Center, Pediatric Department, Meir Medical Center, Sackler School of Medicine, Tel Aviv University, Tel-Aviv 6100000, Israel; Dan.Nemet@clalit.org.il (D.N.); Eliakim.Alon@clalit.org.il (A.E.)

**Keywords:** childhood obesity, gait, postural balance, intervention program

## Abstract

The article reviews the biomechanical factors that may cause overweight/obese children to reduce their level of physical activity, while increasing their risk of overuse injuries and exercise-related pain. Recommendations would be to screen those children for any gait or postural impairments before they join any exercise program, and to provide them with specific gait treatments and/or physical exercise programs, in order to decrease their risk for future musculoskeletal injuries and pain.

## 1. Introduction

Childhood obesity has become a major public health problem [1]. Overweight and obese children present deficient gait patterns [2], insufficient postural control [3], increased forces/impacts applied to the lower extremities [4], lower extremity mal-alignment [5], and reduced bone mineral density, which may lead to increased incidence of overuse injuries and bone fractures [6].

Childhood obesity is mainly associated with reduced physical functioning and disability, which implies a negative impact on daily activities [7]. Intervention programs during childhood may provide an opportunity to improve functioning and decrease disability, to optimize health, and to prevent chronic diseases related to inactivity. The intervention programs should start at a young age, before the onset of obesity-related degenerative musculoskeletal changes that are prevalent in obese adults [8]. Physical activity intervention programs may improve obese children’s gross motor quotient and locomotor skills acquisition; factors which are critical in physical activity participation during later childhood and adolescence, and for reducing their higher risk for overuse injuries and exercise-related pain [8,9].

Yet, multiple musculoskeletal discomfort and pain associated with physical activity may seriously decrease the motivation to exercise and limit the performance of obese children during locomotion [9,10]. When obesity is combined with increased musculoskeletal pain or disorders, the obesity cycle is perpetuated by encouraging sedentary behavior for prolonged periods. Therefore, it is particularly important to identify the associations among childhood obesity, gait pattern, physical activity/inactivity, and intervention programs [11].

## 2. Methods

A search of medical literature databases was conducted for manuscripts published from their inception until March 2018 (Medline/PubMed and SportDiscus). The search was designed to have maximum sensitivity in order to avoid missing any relevant papers. There were four parts to the search: (i) Childhood obesity; (ii) biomechanics (e.g., gait analyses); (iii) musculoskeletal injuries; and (iv) intervention programs. Within each component, MeSH terms and free text descriptors were combined with the Boolean operator ‘OR’, and then the four components were combined with the Boolean operator ‘AND’ (key words—postural balance, locomotion, temporospatial, injuries, mal-alignment, etc.). Search yields were recorded, and the results exported to Endnote. The database search was supplemented by forward and backward citation tracking of the included articles. In the backward citation analysis, the reference lists of all included articles were examined for relevant studies. In the forward citation analysis, all papers that cite the included papers were listed—this list was then reviewed for relevant papers. Finally, the related-articles function within the PubMed was used to identify similar papers for each of the included papers; and this list was reviewed for relevant papers.

Papers were included if they met the following criteria: (i) They concerned obese children or adults; (ii) the subjects had no severe musculoskeletal injuries or had not had recent musculoskeletal surgery; and (iii) the subjects had no medical cause for their obesity (e.g., hypothyroidism) or received any medication that may cause obesity (e.g., corticosteroids). Eligible papers were written in English and published in a peer-reviewed journal, with no limitation regarding publication year. Our exclusion criteria meant letters, conference proceedings, case reports, brief reports, and abstracts were excluded.

## 3. Gait Pattern

Normal gait: Walking is an important activity of daily living, and humans naturally walk at different velocities [12]. Gait is a mode of bipedal locomotion, in which a period of double support (when both feet are in contact with the ground) is followed by a period in which the body is supported by one lower limb, while the other swings forward [13]. Gait pattern is mostly reported by temporospatial parameters, including walking speed, stride time and length, and step time and length, as well as the duration of the stance phase and the swing phase [14]. A step is one single step; while a stride is a whole gait cycle. The step time is the time from one foot hitting the ground to the other foot hitting the ground. Step width can be described as the mediolateral space between the two feet. The stance phase begins with the heel strike—this is the moment when the heel begins to touch the ground, but the toes do not yet touch. In the mid-stance phase, the foot settles along the lateral border, and, during the change from mid-stance to toe-off stance, the five metacarpophalanges contact the ground. The toe-off phase is also named the propulsive phase. When the stance phase ends, the swing phase begins [15].

It is becoming increasingly apparent that childhood obesity is associated with reduced physical functioning and disability, both of which have a negative impact on the walking and running ability of these children [7]. Recently, Oliveira et al. [16] attempted to determine the cutoff points of moderate to vigorous physical activity needed to prevent overweight and obesity. In boys, body mass index (BMI) was found to be negatively associated with the number of steps the children walked per day and with the amount of physical activity. Boys who walked ≤10,502 steps per day had a significantly higher risk of being classified as overweight/obese child [16].

Walking and running skills, like other motor skills, need to be acquired and practiced during early childhood. Both of these natural skills have been described as easy to adopt, with low requirements that can decrease sedentary habits and easily increase the level of physical activity [17,18].

Table 1 shows a number of examples of significant differences in gait pattern between overweight/obese children compared with normal-weight children. When examining gait pattern, overweight children’s walking was described as different and less stable compared with that of their lean counterparts [19,20,21,22]. In general, most studies reported that the gait of overweight children seems to involve longer and wider steps, shorter single-limb support time and longer double-limb support times, shorter swing times, and slower walking velocities compared to normal-weight children [19,20,21,23,24,25,26]. Hills and Parker [27], for example, described the gait pattern of overweight children as characterized by increased step width and a greater percentage of the gait cycle spent in stance. Dufek and colleagues [19] identified a significantly slower walking velocity, lower percent of double support, lower percent of swing phase time, and greater stance width in overweight children compared with their normal-weight counterparts [19].

In a study on running, by Rubinstein et al. [28], elongation of cycle length, increased cycle time, increased stance phase time, increased relative stance phase, and a shorter relative swing phase were found among overweight children compared to normal-weight children; in 80% and 100% of their self-selected preferred running speeds, respectively. In running, similar to walking, the walking speed was found to affect gait elements such as kinematic parameters, foot pressure, and spatio-temporal parameters [23]; increased running velocity (from 80% to 100% self-selected running) increased the cycle length and the relative swing phase among the overweight children [28].

It should be mentioned that most previous comparisons of running patterns between overweight and normal-weight children described the physiological deficits of the overweight children, but not the biomechanical characteristics of their running [40,41]. These physiological studies explained that the running performance of overweight children showed higher oxygen consumption and greater time to recover from exercise compared to that of normal-weight children [40]. The biomechanical changes in gait pattern (walking and running) of the overweight children could be a compensation strategy to reduce the energy cost required to lift, lower, accelerate, and decelerate their excess body mass [24,42,43]. These movement deficits might reduce the energy expenditure during any physical activity of overweight children, and may contribute to an imbalanced energy equilibrium [43,44]. During walking, overweight children might need a higher energy cost compared with their lean counterparts, due to the higher muscle requirements for moving the body segments and maintaining their stability, and for raising and accelerating body weight against gravity. The slower walking of the overweight children, combined with a lower rhythm and a longer double-support phase, might be an adopted walking strategy to avoid the increased metabolic cost and the increased mechanical work, required for moving their excess body mass [24,42,43]. Furthermore, the double support percent of cycle was found to be the most highly predictive of BMI%; hence, longer double support might serve as a compensatory adaptation for a shorter period of single support during walking, which acts to reduce the joint loads from a single-leg support [19].

Another explanation for the different temporal pattern of overweight children compared to their lean counterparts is the reduced postural stability and the muscle weakness of the overweight children [35]. The slower gait velocity, along with reduced postural stability, were explained by the difficulty of overweight children in controlling the fall of the center of gravity, and that it is an adaptive strategy to preserve equilibrium [34]. Muscle weakness, especially around the knee—a joint which is prone to damage and injury, might cause different kinematics of the lower extremities during the walking of overweight children [45]. The additional power requirement without appropriate strength gains could lead to musculoskeletal fatigue, and contribute to the overall poor performance of overweight children during fitness tests [5]. Therefore, it might be suggested that by improving strength and stability in the ankle/foot area, overweight children could improve their gait parameters as well. Furthermore, any improvement in muscle strength might decrease the occurrence of lower extremity pain and discomfort, increase the physical activity level, and lead to improved fitness of overweight children [46].

Recently, Hung and colleagues [29] explained that overweight and obese children organize their whole body movement during a simple pick-up task differently than normal-weight children. Their movement strategy may put them in a less stable condition, and thus make them prone to losing balance. Other studies suggest that the dual task condition is more challenging for the overweight and obese group than for the normal-weight group [47]. During a box-carrying task (dual task condition), the overweight and obese children decreased their movement velocity even more than in their simple walking condition. These findings suggest that decreasing velocity could help compensate for the increased attentional demands required for completing activities with dual task constraints. The overweight children should be aware of their movement control difficulties during everyday movements, for their safety [29,47].

## 4. Impact/Loads to the Lower Extremities and the Lower Back

Repeated durations of dynamic activity with increased impact to the lower extremities and the lower back can be beneficial to bone health, although this could also cause potential overuse injuries [33,48]. Measurements of the impact to the lower limbs are mainly evaluated during gait analysis, in parameters such as ground reaction forces (measured during the foot contact phase, to obtain the speed and vertical displacements of the center of body mass), peak acceleration (peak positive acceleration of the tibia), dynamic plantar pressure (providing information on dynamic loading to the lower limb), and others [32,41,49].

In the general population, subjects with a history of tibial stress fracture were reported to have a high loading rate of ground reaction forces, and a higher peak positive acceleration of the tibia than those without this medical history [48]. Reducing the loading rate of ground reaction forces by 10–15% was suggested for prevention and treatment of the stress fracture. Furthermore, monitoring the peak positive acceleration during gait (by feedback, for example) can provide substantial information on gait quality, and may reduce the risk of lower extremity injury [49]. Considering children, Tirosh et al. [49] analyzed peak positive acceleration of the tibia at different gait speeds in children aged 7–12 years old, and reported a significant increase in peak positive acceleration with increased gait speed [49].

Measuring the impact (ground reaction forces, peak positive acceleration, or dynamic peak pressure) to the lower extremities and lower back among obese children can be useful for detecting their risk for injuries [50,51]. Yet only a limited number of studies have measured the impact of these in overweight children and adolescents during walking and running [30,33,52], with quite similar results and conclusions in those studies. It was reported that the obese children exerted greater ground reaction forces in the medial-lateral and anterior-posterior directions, and less ground reaction forces in the vertical direction, compared to children of normal body weight [53]. The plantar pressure measurements (such as contact area, peak pressure, and force time integral) were found to be higher, especially in the mid-foot area, during the gait of obese children compared to that of normal-weight children [31,32]. The continual bearing of excess mass was found to flatten the mid-foot region during walking, with higher dynamic plantar pressures in the mid-foot and forefoot regions, compared to the pressures in normal-weight children [27,52,54,55]. Significantly larger contact areas and larger forces on the plantar surface, as well as increased peak pressures, increased peak forces, and higher peak area under the fore-foot, mid-foot, and lateral heel, were found in overweight children during walking compared to normal-weight children [28,30,32,33]. In running, similar to walking, parameters such as peak pressure, maximum force, pressure time integral, and force time integral increased with increasing running velocity, and were higher in overweight compared with normal-weight children [28]. When a backpack was carried by the overweight/obese children, altered plantar pressures similar to that observed in normal-weight peers were found. Yet, the pressures were much higher among the overweight/obese children compared with the lean children. This raises concerns regarding potential long-term adverse consequences on foot structure and functionality, and supports establishing more specific limits for the carried load [56].

Obese children are incapable of compensating for the extra body mass that leads to an equal plantar load distribution across all foot regions, compared to non-obese children. The fact that the peak pressure mostly affected the mid-foot and forefoot, and to a lesser extent the rear foot in these children, might be an adaptation strategy to compensate for their additional body weight [31]. The hip joint of obese children might also be at higher risk for injuries, due to greater compressive and shear contact forces and loading rates compared to normal-weight children during walking [57].

Most studies suggest that the increased pressure/loads in obese children may increase loading on the developing foot, and may result in foot discomfort and possibly deformity, which may increase the risk of pain and injury [30,33,38,52,54,55,58]. As prepubescent children’s feet continue to develop and mature, some of the higher loads/pressures during more vigorous weight-bearing locomotor activities, such as running, might be associated with an even greater mass bearing on the developing feet, which may increase the risk for foot pain and foot dysfunction [59]. In addition, a few studies reported that the high loads/pressures along the lower extremities of overweight children were severe enough to reduce their participation in physical activity programs, especially when they were required to run [30,33,59]. This general pattern of increased impact/loads among obese children has caused some clinicians to weigh the risk-benefit ratio of increased physical activity on the musculoskeletal system in obese children [57]. Nevertheless, most previous studies recommended intervention programs for both weight reduction and for reduced loads/impacts to the musculoskeletal system [30,33,57]. As the increased impacts/loads on the overweight child’s feet may occur when they are very young, early assessment and intervention is required in these children to mitigate the development of musculoskeletal complications associated with excessive body mass [60]. It is possible, however, that screening for increased musculoskeletal and particularly foot impact/loads prior to participation in weight-management exercise programs would identify those obese children at risk, so that necessary precautions can be taken to improve their participation in such programs and reduce the risk of overuse injuries.

Table 1 shows some numerical examples of significant differences between overweight/obese children and normal-weight children in the impact/loads to the lower extremities and the lower back.

## 5. Postural Balance

Maintaining a stable posture is essential for many daily activities, as well as for injury prevention. Postural balance is the act of achieving, maintaining, or restoring a state of balance during any posture or activity [61]. Most daily activities involve components of static balance, as well as complex dynamic movements [62]. Static postural balance is the ability to maintain a base of support with minimal movement, whereas dynamic postural balance reflects the ability to perform a task while maintaining or regaining a stable position [62]. Postural balance is controlled by using perceptual information obtained from the environment by the peripheral sensory systems [63]. Maintenance of postural balance requires an integration of the visual, vestibular, and somatosensory systems, in order to coordinate the sensory perception of the body’s position and execute motor responses [61,64].

Among children, several factors may influence postural balance, including age, gender, and fitness level [59,65]; however, the influence of weight on postural balance in children is not completely understood [36]. It has been well documented that excessive body weight is inseparably connected to postural instability and to body weight distribution [66]. The mass of the pendulum in obese children is high, causing the muscles to generate torques of higher amplitude in order to maintain balance. As obese children mostly suffer from decreased muscle tissue relative to the increased body weight, their muscles often cannot respond quickly and strongly enough to the higher center of pressure (CoP) displacement. The increased magnitude of the CoP combined with relatively weak muscles might increase the risk of falling in obese children [36,56,67].

Several studies have suggested that obesity imposes significant constraints on balance control [6,36]. For examples of significant differences between overweight/obese children and normal weight children, see Table 1. It has been noted in the literature that compared to normal-weight children, obese children have lower postural stability [3,34,35], greater sway area with greater instability [36,54], and different postural strategies with reduced balance capabilities [56]. Due to the higher CoP speed and greater postural instability of obese children, the time it takes them to correct their movements during different tasks is much longer [68]. Furthermore, there is general agreement that compared with normal weight children, obese children have a higher prevalence of perceived clumsiness [69], coordination difficulties [70], and decreased motor skills [70]. The obese children manifested decreased fine motor skill performance, mainly due to underlying perceptual-motor coordination difficulties, as compared to their lean age-matched controls [6].

As the increased mass affects the mechanical and sensorial systems involved in postural control, the central nervous system has to adapt its control actions to maintain balance [67]. Taking into consideration the three systems controlling postural balance, removing visual feedback (e.g., standing with closed eyes) dramatically reduced the postural balance of obese children compared to non-obese children [35,54], although the natural tendency of all children (regardless of their weight) is to increase postural sway with closed eyes [71]. Yet, in order to counterbalance their decreased postural sway, obese children might be more dependent on vision than leaner children [54].

Another system that might be less effective in obese children is the somatosensory system [67]. The excessive pressure on the obese children’s feet might alter the activity of the plantar cutaneous sensory receptors; this may reduce the sensory feedback required to coordinate the body’s position and to maintain postural balance [67,72,73].

### Falling Risk

Poor postural balance in obese children may be associated with more frequent falls and with a higher risk of fractures [37,74]. As explained earlier, the increased mass forces the muscles to work harder in order to maintain balance. However, if the muscles cannot respond quickly and strongly enough, there is a high risk of falling [67].

In Steinberg et al. [35] it was found that 40% of the obese children had a moderate to severe falling index (assessed by the Posturography device, which calculates the risk of falling with a proprietary algorithm) compared to normal-weight children’s norms. Although some studies indicated that overweight and normal-weight children have the same chances of falling [3], most studies reported that obese children have a higher risk of falling and fractures than leaner children [36,63,75,76]. As discussed above, obese children demonstrated different gait patterns, with increased foot pressure as compensation for their postural instability and as an attempt to slow themselves down in order to avoid falling [34,54]. In addition, as obese children have a decreased ability to control falls compared with their lean counterparts, it is expected that obese children will fall more frequently, with more force, and in more awkward positions, which may increase the risk for fractures during their everyday activities [6,69].

## 6. Malalignments and Injuries

Compared with normal-weight children, overweight children and adolescents were reported to suffer more often from musculoskeletal discomfort, orthopedic deformities (such as scoliosis and lumbar hyper-lordosis), skeletal diseases (such as osteoarthritis), injuries (e.g., bone fractures), structural changes of the plantar surface, reduction in flexibility, and malalignment of the lower extremities [5,9,37,38,74] (see Table 1). Early diagnosis of lower-extremity malalignment seems to be crucial in obese children, in order to prevent future injuries/diseases such as osteoarthritis [77].

### 6.1. Fractures and Other Types of Injuries

Musculoskeletal pain was found to be more prevalent in overweight children and adolescents compared with normal-weight children [37]. Over 60% of the obese children complained of at least one joint (mostly the back, foot, or knee joint) pain more than once per month [78], with persistent pain and swelling after injuries such as ankle sprain [79].

Considering injuries, Witt et al. [39] showed that children with higher BMI percentiles had a significantly higher prevalence of extremity injuries. Obese and overweight children were reported to have 25% more extremity fractures than non-obese children [80]. Most overweight children present increased bone mineral density and increased bone cross-sectional area [81,82,83]. The greater bone mineral density found in overweight children was expected to protect against fractures [81]. In contrast, obese and overweight children had no difference in bone strength compared to lean children [84], and even presented reduced bone strength, as determined by quantitative ultra-sound measurements of bone speed of sound [85]. It might be argued that even an increased bone mineral density of overweight children might not be sufficient enough to protect their bones from the significantly greater forces and impact (caused by a fall with higher body weight) that are generated during falls [81,86]. The lower foot sensitivity of obese children, due to the higher plantar pressure, was also found to be a risk factor for a variety of foot injuries [87]. In addition, the fact that the overweight children had impaired mobility may contribute further to their increased risk for falls and fractures [36,88]. It should be mentioned that overweight children mostly tend to fall from lower heights (as they avoid rapid-movement activities and climbing activities), yet they exhibit a greater risk for fractures [88,89].

### 6.2. Malalignment

Early childhood obesity might be a particular risk factor for malalignment, and consequently for orthopedic complications. The increased joint loading and joint forces before and during puberty place the growing skeleton of the obese child under continuous pressure along that critical growth-spurt period [90]. In the development of the lower extremity axis, greater vertical loads/pressure may lead to abnormal bone growth and formation, causing malalignments and joint deformities [37]. Obesity was found to negatively impact the foot structure, the lumbar spine, and the lower extremity joints (such as in genu valgum and genu recurvatum) [77]. Around the knee joint, for example, Gushue et al. [91] and Strutzenberger et al. [92] found that obese children have greater knee moments, causing greater loading of the musculoskeletal structures compared to normal-weight children, which may cause some bone deformities and malalignments, such as knee valgus [93]. Overweight/obese children were also reported to have a significantly higher prevalence of flat foot [94] compared with lean children. The lower plantar arch height caused by structural changes in their foot anatomy, as a result of excess weight-bearing, continues throughout childhood and into adulthood [38,91,92]. Any malalignment in overweight and obese children may lead to skeletal discomfort and pain [37].

Malalignment of the knee might be a risk factor for osteoarthritis [90,95,96]. Similar to obese adults, where approximately 50% have knee cartilage lesions [97], obese children and adolescents suffering from knee pain also showed morphological changes in their knee cartilage that may develop into osteoarthritis later in life [98].

## 7. Intervention Programs

The different biomechanical factors reported in overweight children can be particularly important when those children join exercise training programs, or increase their physical activity in an effort to maintain or reduce body weight (see Table 2) [11,22,27,42,72,99,100,101,102,103,104,105,106,107,108]. As reported earlier in the current review, overweight children have a different gait pattern, increased impact/loads to the lower extremities, increased prevalence of joint malalignment, and postural balance deficits, compared with normal-weight children. Any intervention exercise programs should be carefully designed in an attempt to improve their gait pattern and their postural balance, and in trying to minimize their risk of falls to prevent future musculoskeletal injuries and pain [9,17,18,46,103].

While interventions to improve movement skills in early childhood (pre-school children) appear to be effective [109,110], less is known about their impact on movement characteristics (such as gait pattern) in overweight school-aged children. Most intervention programs for school-aged children focus on weight reduction and reinforcing healthy nutrition and eating habits, aiming at reducing media use but not on improving biomechanical function [111,112,113,114]. Up to now, only a few studies have examined the effect of strength training and neuromuscular exercise interventions on walking and movement patterns, and on biomechanical gait characteristics of overweight children [22,100,106,108].

**Figure 1:**
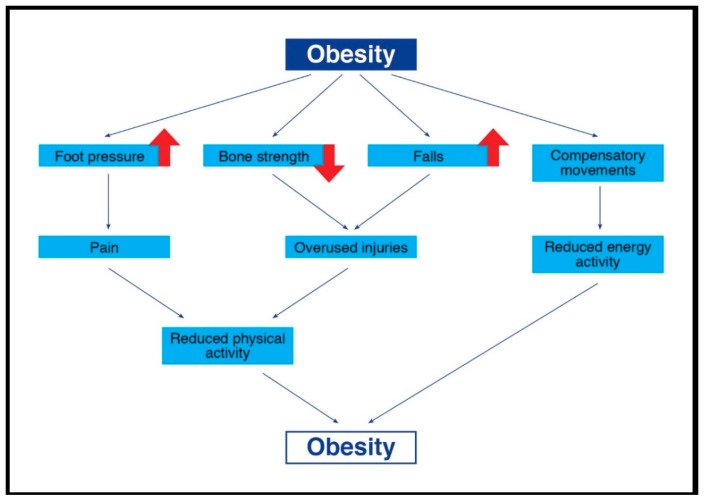
Vicious cycle of childhood obesity.

In a previous study [22], it was indicated that overweight children who participated in a locomotion-emphasis program improved their gait pattern and decreased their foot loading during both walking and running, when compared with overweight children who had no intervention programs. The authors suggested that early assessment and intervention for improved gait and for decreasing foot pressure among overweight children be required in order to reduce the risk of developing musculoskeletal complications associated with excessive body mass [22]. In another study, both weight-loss and muscle-strength training led to positive changes in gait kinematics and kinetics among overweight children; however, the temporal gait parameters of the overweight children did not change following weight loss [11]. It was also found that obese children participating in physical activity intervention programs improved their postural balance, yet their postural balance following the intervention program was still lower than their normal-weight counterparts [108]. Recently Han and colleagues [115], in their systematic review, showed that overweight/obese children have lower levels of fundamental movement skills than normal-weight children. However, interventions are effective in improving their skills. The authors recommended these physical exercise intervention programs in order to break the vicious cycle of childhood obesity (Figure 1).

Finally, the impact/forces to the lower limbs of obese subjects were found to be inversely related to lean mass, as well as to a low lean mass/fat mass ratio that could contribute to early leg muscle fatigue. When considering intervention programs, it would be beneficial not only to reduce body weight but also to increase lean the mass/fat mass ratio in obese children. Obese children need to be encouraged to take part in intervention programs that include physical exercise in order to lose weight and also to prevent lower extremity injuries [20].

## 8. Conclusions and Clinical Implications

**Figure 2:**
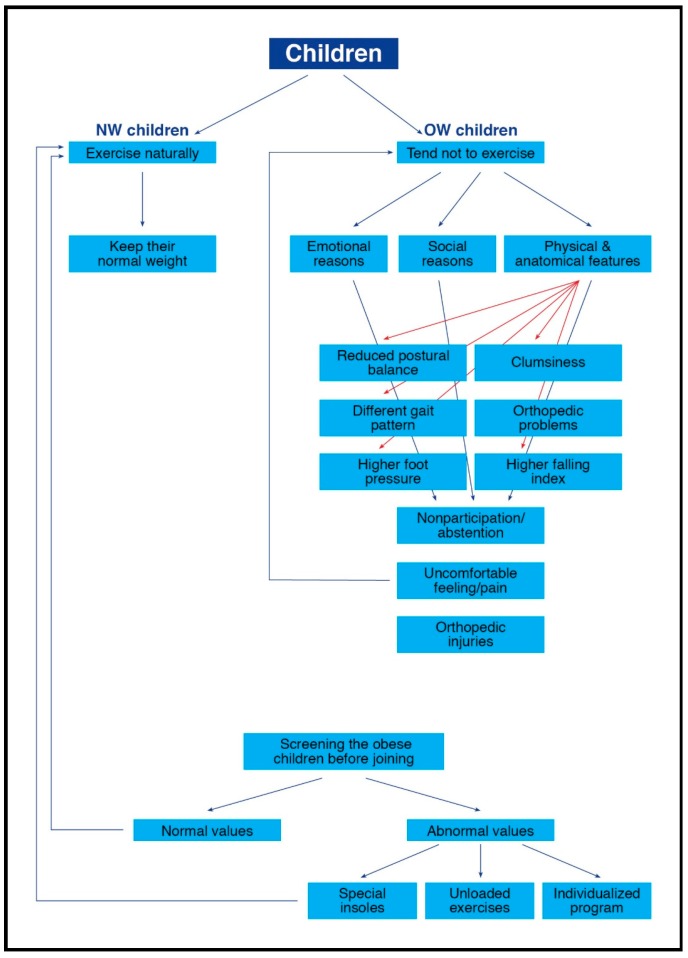
Screening the OW children.

A relatively high proportion of children with overweight and obesity exhibit impairment of balance and gait patterns, increased foot pressure, and reduced bone strength, and as a result they have an increased susceptibility to overuse injuries and the tendency to withdraw from weight management programs that involve physical activity. Therefore, we believe that before overweight/obese children and adolescents join any physical activity weight-management program, the following parameters should be considered (see Figure 2):

(1) Gait analyses: These analyses should identify gait impairments. If such deficiencies exist, these children should participate in a specific program, such as a locomotion-emphasis program for improving biomechanical characteristics [28], prior to participation in the weight-management intervention. This program should focus mainly on improving the ankle-foot gait movements (e.g., exercises for improving ankle mobility and stability, improving postural balance, improving proprioception ability, and strengthening muscles such as the calf muscles and the extrinsic and intrinsic foot muscles).
(2) Impact/loads to the lower extremities and the lower back: As increased impact/loads might increase the chance for foot malalignment and musculoskeletal injuries, interventions such as visual biofeedback gait retraining should be advised for these children. Furthermore, in order to decrease plantar pressures beneath the feet, a potential implication for innovative children’s footwear design is suggested [46,87].
(3) Postural balance assessment: Safety measures to identify the overweight/obese children who suffer from postural balance deficiencies might decrease their chances of falling and of subsequent injuries during participation in physical activities. It is important that the initial phases of exercise interventions focus on balance improvement (using exercise on stable and unstable surfaces, with open and closed eyes, static and dynamic, etc.) [108].
(4) Bone strength: Bone strength should be determined in order to detect overweight/obese children with reduced bone properties [85]. Children with reduced strength and increased fracture risk should start intervention programs with non-weight-bearing exercises, such as bicycle riding or swimming [37], and gradually increase the amount of weight-bearing exercises.
(5) Mal-alignments and previous injuries: As mal-alignments and previous injuries may affect the overweight/obese children’s performance and increase the risk for new injuries, it is suggested that a clinical orthopedic examination should be undertaken before starting an exercise program [87]. Individualized and gradually adapted exercise program should be implemented for each child.

## Figures and Tables

**Table 1:** Examples for significant differences between overweight/obese children compared with normal weight children in gait pattern, impact/loads to the lower extremities and the lower back, postural balance and malalignments, and injuries.

Authors	Gr.	Age	BMI	Measured Parameter	Results	
**Gait Pattern**						
Dufek et al. [19]	OW	14.9 ± 1.2	95.5%	Custom walkway software was used to visually inspect walkingTrial performed for completeness	Double support percent of cycle	24.0 ± 3.1
						20.2 ± 3.3
					Swing phase percent of cycle	38.1 ± 2.0
	NW	14.7 ± 1.5	57.9%			40.0 ± 1.2
					Heel to heel stance width	11.2 ± 3.5
						8.8 ± 2.6
Rubinstein et al. [28]	OW	9.9 ± 1.3	24.3 ± 3.5	Temporal parameters measured for 120% of the self-selected preferred walking speeds	Cycle length	1.3 ± 0.1
						1.1 ± 0.18
	NW	9.9 ± 1.2	17.1 ± 2.0		Cycle time	0.8 ± 0.1
						0.7 ± 0.1
					Stance phase time	0.5 ± 0.1
						0.4 ± 0.0
Hung et al. [29]	OW	8.2 ± 0.3	22 ± 4.6	Anterior/posterior center of pressure measurements during picking up an empty box to waist height at a self-selected pace	Normalized COP Ant/post Excursion	8.5 ± 1.5
						7.0 ± 1.4
					Speed of COP Move Anterior	16.7 ± 4.3
						12.2 ± 3.5
					COP Kept Anteriorly Time	0.3 ± 0.1
						0.01 ± 0.0
Yan et al. [30]	OW	9.6 ± 1.6	23.7 ± 3.0	Gait data (LT) such as arch index and foot balance parameters	Arch index	0.32 ± 0.06
						0.28 ± 0.04
					Midfoot Relative regional impulse	7.01 ± 3.35
						5.56 ± 2.08
	NW	10.3 ± 0.7	17.1 ± 1.3		Maximum Heel strike phase	13.8 ± 8.6
						20.1 ± 11.7
					Maximum Mid- stance phase	7.7 ± 7.7
						19.6 ± 10.4
					Propulsion phase	10.8 ± 8.9
						16.8 ± 12.7
**Impact/loads to the Lower Extremities and the Lower Back**						
Rubinstein et al. [28]	OW	9.9 ± 1.3	24.3 ± 3.5	Foot pressure parameters in lateral forefoot area in 120% of the self-selected preferred walking speeds	Contact area	4.2 ± 0.9
						3.4 ± 1.2
	NW	9.9 ± 1.2	17.1 ± 2.0		Peak pressure	172.5 ± 46.9
						108.8 ± 42.7
Mueller et al. [31]	OB	7.2 ± 3.2	23.1 ± 3.3	Peak pressure measurements	Force time integral in midfoot	22.4 ± 19.4
						7.9 ± 6.7
	NW	7.0 ± 2.8	16.4 ± 1.5		Peak pressure (total foot) aged 12	512 ± 177
						409 ± 124
Mickle et al. [32] 2006	OW	4.5 ± 0.8	18.6 ± 1.3	Plantar pressures were assessed to characterize dynamic foot function	Force-time integrals in midfoot	10.2 ± 5.6
						5.5 ± 4.1
	NW	4.5 ± 0.7	5.8 ± 0.7		Pressure-timeIntegral in midfoot	2.0 ± 0.7
						1.6 ± 0.5
Dowling et al. [33]	OW	8.1 ± 1.2	>95%	Dynamic plantar pressure assessment with the subjects loaded with an additional 20% of their body mass	Static peak force	394.3 ± 112.1
						278.1 ± 52.6
					Static peak area	72.6 ± 17.3
						51.5 ± 7.6
					Dynamic peak force	558.9 ± 119.3
						365.6 ± 61.8
					Dynamic peak area	101.1 ± 12.0
						78.1 ± 9.9
					Dynamic rear foot force	399.2 ± 91.9
						260.3 ± 38.4
					Dynamic rear foot area	39.3 ± 6.4
						25.1 ± 5.0
					Dynamic forefoot force	515.3 ± 89.5
						354.4 ± 60.8
					Dynamic forefoot area	50.0 ± 5.7
						41.3 ± 4.1
Yan et al. [30]	OW	9.6 ± 1.6	23.7 ± 3.0	Dynamic plantar pressure distribution: sub-phases during foot-ground contact duration (LT)	Midstance phase duration	49.5 ± 7.7
						43.1 ± 10.6
	NW	10.3 ± 0.7	17.1 ± 1.3		Propulsion phase	41.8 ± 7.8
						46.2 ± 9.2
**Postural Balance**						
Deforche et al. [3]	OW	9.3 ± 1.0	23.8 ± 3.1	Balance Master, a computerized pressure plate system	Weight transfer time	0.45 ± 0.60
						0.21 ± 0.16
					Rising index	36.2 ± 7.9
						44.8 ± 12.3
					Centre of gravity sway velocity	5.1 ± 0.9
						4.1 ± 1.1
	NW	9.3 ± 0.8	16.3 ± 1.2		Steps width	22.1 ± 3.0
						19.5 ± 3.4
					Unilateral stance dominant leg	8.9 ± 1.8
						10.0 ± 0.0
					Heel-to-toe walk	3.7 ± 1.8
						5.6 ± 1.2
					Five times up and down	10.3 ± 1.3
						8.6 ± 2.0
Colne et al. [34]	OW	Adolesc.	40 ± 5	Postural stability and gait initiation	Double support	158 ± 26
						142 ± 19
					Length of first step of gait initiation	0.85 ± 0.13
						0.87 ± 0.07
	NW	Adolesc.	20 ± 2		Peak of the post-ant velocity of CG	1.36 ± 0.19
						1.65 ± 0.09
					Mean velocity of the CP	1.69 ± 0.33
						1.83 ± 0.21
Steinberg et al. [35]	OW	6–128.8 ± 1.7 G9.6 ± 1.9 B	92% OB8% OV	Low Falling Index (FI) ≤ 36 points, moderate FI = 36–40 points, severe FI ≥ 41points	Mean FI for NW = 36 points	Mean = 28.2FI
						27.6% mod. FI
						12% severe FI
Goulding et al. [36]	OW	14.8 ± 2.4	21.4 ± 4.2	The Bruininks/Oseretsky sub-test of balance; the Equitest sensory organization test; and Balance Master limits of stability test	Bruininks/Oseretsky composite score	24.5 ± 3.2
						26.6 ± 2.5
					Equitest SOT score *	74.6 ± 7.0
						73.7 ± 8.4
					Equitest SOT score *	0.65 ± 0.21
	NW	14.9 ± 2.4	19.6 ± 2.6			0.67 ± 0.25
					Movement velocity *	5.66 ± 2.22
						5.35 ± 1.98
					Directional control *	73.2 ± 8.5
						75.0 ± 9.1
**Malalignments and Injuries**						
Taylor et al. [37]	OW	12.6 ± 2.7	36.4 ± 8.9	Malalignment: metaphyseal-diaphyseal angle (MDA) and anatomic tibiofemoral angle (A-TFA)	MDA (RT)	−6.5 ± 4.6°
						−5.2 ± 3.9°
	NW	11.8 ± 2.9	19.6 ± 3.5		Abnormal MDA and A-TFA	>11%
						<3.2%
Mickle et al. [38]	OW	4.3 ± 0.9	18.6 ± 1.2	Foot anthropometry	Arch index	0.26 ± 0.05
						0.20 ± 0.9
	NW	4.3 ± 0.7	15.7 ± 0.7		Plantar arch height	0.9 ± 0.3
						1.1 ± 0.2
Witt et al. [39]	OB	11.9 ± 4.7	97.3 ± 1.2%	Operative interventions for patients with severe injury to body region	Femur fixation	47.8%
						45.0%
	NW	12.2 ± 5.4	51.3 ± 23.7%		Intensive care unit, length of stay	4.5 ± 6.9
						4.0 ± 5.7

* No Significant differences between OW and control group (*p* > 05). GR = group; Adolesc = adolescents OW = overweight and obese children; OV = overweight; OB = obese children; NW = normal weight; G = girls; B = boys.

**Table 2:** Intervention programs for childhood obesity.

Authors	BMI	Age	Type of Intervention	Duration of Intervention	Main Findings
**Gait Pattern**					
Huang [1]	30.2 ± 3.3	10.7 ± 1.1	Weight loss intervention (fun-based exercise, nutrition and behavior education)	4 weeks	Weight loss with reduced body circumferences causes mass-driven changes in joint kinematics and kinetics; yet, the spatiotemporal gait parameters did not change
	26.5 ± 1.5	10–12 years	Muscle strengthening (dynamic, resistance exercises)	8 weeks	Significant increases in absolute and relative muscle strength of the lower extremities compared with controls
Huang et al. [101]			Exercise weight-loss program	4 weeks	Reduce stance phase after weight loss
Peyrot et al. [42]	M: 32.0 ± 3.9 F: 36.5 ± 5.4	12–16 years	Weight reduction program, including nutritional education, caloric restriction, and physical activities	12 weeks	Increased stride length with less leg muscle work to raise the center of mass after weight loss
Steinberg et al. [22]	96.99 ± 2.14 percentile	9.4 ± 0.8	Multidisciplinary program with locomotion-emphasis exercises	6 months	Improved foot pressure (at the heel, medial midfoot, lateral midfoot, and lateral forefoot) Improved temporal parameters (cycle length, stance phase time, relative stance phase, and swing phase time)
**Postural Balance**					
Kuni et al. [103]	F: 25.2 ± 3.6 M: 26.2 ± 2.8	6–12 years	“Ball School Heidelberg”—A basic introduction to ball games for school children	6 months	Ball games and nutrition counseling improved postural control
Steinberg et al. [108]	96.9 ± 2.3 percentile	6–14 years	weight management program (including dietary intervention and exercise program)	6 months	Improved postural stability and decreased falling probability
Physical Fitness and Muscle Strength					
Benson et al. [99]			High-intensity progressive resistance training	8 weeks	Improvement in central and whole body adiposity in association with improved muscle strength
D’Hondt et al. [72]	29.1 ± 3.6	10.5 ± 1.4	Multidisciplinary residential obesity treatment program including gross motor and co-ordination exercises (assessed using the Körperkoordinationstest für Kinder—KTK)	4 months	Treatment was found to be efficacious in generating a significant progress in gross motor co-ordination performance, with a greater increase in KTK score The amount of relative weight loss explained 26.9% of the variance in improvement in overall KTK performance
Horsak et al. [100]	> 97th percentile	10–18 years	Muscle strength training and neuromuscular exercises	12 weeks	-
Korsten-Reck et al. [102]	> 97th percentile	8–12 years	FITOC (Freiburg Intervention Trial for Obese Children) consists of a combination of organized sports, behavioral therapy and nutritional advice	8 months	Performance in all motor abilities tests improved (The AST-test battery included two speed tests, one aerobic capacity test, two strength tests and three coordinative tests) The difference between the strength of the obese children and the strength of the reference group decreased
Larsen et al. [104]	24.8 (22.8–27.1)	12.0 ± 0.4	Two groups: Day-Camp Intervention (DCIA), with a subsequent family-based support program; and low-intense Standard Intervention Arm (SIA)	52 weeks	Balance skills were improved post-camp in DCIA group compared to the SIA Children from the SIA improved motor skills relative to the DCIA children
Morano et al. [105]	≥95th percentile	9.2 ± 1.2	Multi-component treatment program focused on attaining a physically active lifestyle by increasing their actual and perceived competence in performing motor tasks	8 months	Gross motor performance (such as squat jump and countermovement jump) and actual and perceived physical abilities (perception of strength, speed and coordinative abilities) significantly improved 8 months after treatment in obese boys and girls
Sola et al. [107]	27.4 (24.8–29.3)	11.5 (9.0–12.5)	Physical fitness with motor abilities (such as balance, speed, agility, coordination and strength)	6–12 months	All physical fitness abilities improved over the intervention period
**Walking Energetics**					
Peyrot et al. [42]	34.6 ± 5.1	12–16 years	Obesity management program	12 weeks	After weight loss, the increased walking economy was induced by the lower metabolic rate of the isometric muscular contractions required to support the lower body weight and maintain balance during walking
Hills & Parker [27]	-	-	Exercise and diet intervention	16 weeks	More stable and symmetrical gait pattern (better symmetry indicators, step length and relative step); improved body composition.
Morrison et al. [106]	13% of all children who participated were overweight and 7% were obese	9–11 years	A do- based PA intervention that were motivated and supported to increase the frequency, intensity and duration of dog walking using a number of behavior change techniques	10 weeks	Using pet dogs as the agent of lifestyle change in PA interventions in children and their parents is both feasible and acceptable
